# AmodalAppleSize_RGB-D dataset: RGB-D images of apple trees annotated with modal and amodal segmentation masks for fruit detection, visibility and size estimation

**DOI:** 10.1016/j.dib.2023.110000

**Published:** 2023-12-30

**Authors:** Jordi Gené-Mola, Mar Ferrer-Ferrer, Jochen Hemming, Pieter van Dalfsen, Dirk de Hoog, Ricardo Sanz-Cortiella, Joan R. Rosell-Polo, Josep-Ramon Morros, Verónica Vilaplana, Javier Ruiz-Hidalgo, Eduard Gregorio

**Affiliations:** a Efficient Use of Water in Agriculture Program, Institute of AgriFood Research and Technology (IRTA), Fruitcentre, Parc Científic i Tecnològic Agroalimentari de Gardeny (PCiTAL), 25003 Lleida, Catalonia, Spain; b Research Group in AgroICT& Precision Agriculture - GRAP, Department of Agricultural and Forest Sciences and Engineering, Universitat de Lleida (UdL) – Agrotecnio-CERCA Center, Lleida, Catalonia, Spain; c Wageningen University and Research, 6700 AA Wageningen, the Netherlands; d Department of Signal Theory and Communications, Universitat Politècnica de Catalunya, Barcelona, Catalonia, Spain

**Keywords:** Instance segmentation, Modal segmentation, Amodal segmentation, Yield prediction, Depth image, Fruit measurement, Fruit visibility, Agricultural robotics

## Abstract

The present dataset comprises a collection of RGB-D apple tree images that can be used to train and test computer vision-based fruit detection and sizing methods. This dataset encompasses two distinct sets of data obtained from a Fuji and an Elstar apple orchards. The Fuji apple orchard sub-set consists of 3925 RGB-D images containing a total of 15,335 apples annotated with both modal and amodal apple segmentation masks. Modal masks denote the visible portions of the apples, whereas amodal masks encompass both visible and occluded apple regions. Notably, this dataset is the first public resource to incorporate on-tree fruit amodal masks. This pioneering inclusion addresses a critical gap in existing datasets, enabling the development of robust automatic fruit sizing methods and accurate fruit visibility estimation, particularly in the presence of partial occlusions. Besides the fruit segmentation masks, the dataset also includes the fruit size (calliper) ground truth for each annotated apple. The second sub-set comprises 2731 RGB-D images capturing five Elstar apple trees at four distinct growth stages. This sub-set includes mean diameter information for each tree at every growth stage and serves as a valuable resource for evaluating fruit sizing methods trained with the first sub-set. The present data was employed in the research paper titled “Looking behind occlusions: a study on amodal segmentation for robust on-tree apple fruit size estimation” [1].

Specifications Table

**Table:** 

Subject	Agronomy and Crop Science, Horticulture, Agriculture Engineering, Computer Vision and Pattern Recognition.
Specific subject area	Precision Agriculture, Fruit Detection, Fruit Size Estimation, Agricultural Robotics, Machine Learning, Deep Learning
Data format	Raw images: JPG Processed RGB images: JPG Processed depth images: NPY (numpy) Modal and amodal segmentation masks: JSON Fruit size ground truth: TXT Images focal length: TXT
Type of data	RGB-D images; modal and amodal segmentation masks; fruit size ground truth.
Data collection	Fuji and Elstar apple trees were imaged using a Canon EOS 60 DSLR and a Nikon Z6 camera respectively. All Fuji apples were manually measured with a Vernier calliper and linked to an apple ID, while 15 Elstar apples per tree were measured providing mean tree diameters. Depth images were generated via Structure-from-Motion (SfM) and Multi-View-Stereo (MVS) using Agisoft Metashape. A total of 3925 image patches of 1024 × 1024-pixel were annotated with modal and amodal masks linked to the apple IDs. Modal masks were annotated using VIA annotator software. Amodal masks were generated based on the apples' spherical shape and diameter-to-pixel conversion using the pinhole camera model.
Data source location	Fuji Data: Agramunt, Catalonia, Spain (latitude: 41° 44′ 47.07′’ N; longitude: 1° 01′ 52.23′’ E; 312 m a.s.l. ETRS89) Elstar Data: Randwijk, The Netherlands (latitude: 51° 56′ 18.25′’ N; longitude: 5° 42′ 24.33′’ E; 312 m a.s.l. ETRS89)
Data accessibility	Repository name: Dataverse Data identification number: https://doi.org/10.34810/data916[2] Direct URL to data: https://dataverse.csuc.cat/dataset.xhtml?persistentId=doi:10.34810/data916
Related research article	Looking behind occlusions: A study on amodal segmentation for robust on-tree apple fruit size estimation. Gené-Mola J, Ferrer-Ferrer M, Gregorio E, Blok PM, Hemming J, Morros JR, Rosell-Polo JR, Vilaplana V, Ruiz-Hidalgo J. 2023. Computers and Electronics in Agriculture 209 (2023) 107,854. DOI: https://doi.org/10.1016/j.compag.2023.107854[1]

## 1 Value of the Data

• **Groundbreaking Amodal Segmentation:** This dataset is pioneering by being the first publicly available dataset for on-tree fruit detection and sizing that includes amodal segmentation masks ground truth. The inclusion of amodal masks, covering both visible and occluded apple regions, offers a unique advantage for on-tree fruit sizing even in the presence of partially occlusions. This feature enables the development of robust automatic fruit sizing methods and accurate fruit visibility estimation, filling a gap in existing datasets.
• **Benefits Across the Agricultural Ecosystem:** The use of this data will benefit researchers in the field of computer vision applied to fruit monitoring. Consequently, this will also benefit the rest of stakeholders of the value chain, such as technology companies offering fruit monitoring solutions, as well as agricultural consultants and farmers that need to obtain fruit production maps and monitor the fruit growth to manage the orchards efficiently.
• **Algorithm Development and Reproducibility:** The provided dataset will facilitate the development and training of modal and amodal segmentation algorithms capable of detecting both visible and occluded parts of apples. The dataset is prepared to be applied to train and fine-tune existing instance segmentation neural networks such as Mask R-CNN [3]. Additionally, the dataset includes ground truth fruit sizes, enabling the implementation and reproduction of results obtained in the fruit sizing methods proposed in [1] and [4].
• **Rich and Diverse Dataset:** With an extensive collection of RGB-D images featuring both modal and amodal segmentation masks, researchers can delve into advanced fruit detection and sizing techniques. The dataset encompasses apples at different maturity stages from both Fuji and Elstar orchards, enabling comparative studies across different apple varieties and growth stages. This multi-orchard perspective broadens the dataset's applications and provides a comprehensive context for researching fruit detection and sizing algorithms.

## 2 Background

The development of automatic fruit sizing methods is of interest for orchard management, with applications in fruit thinning, yield prediction, mapping, and automated harvesting [5]. Traditional manual measurements using Vernier callipers are limited in precision and efficiency, prompting the exploration of image-based approaches. Currently, many image-based fruit sizing methods use modal instance segmentation methods to predict the visible regions of fruits and subsequently measure their size [6]. However, these methods encounter challenges in effectively handling partially occluded fruits. Recently, the authors proposed a solution to this challenge introducing the use of amodal instance segmentation, predicting both visible and occluded regions of fruits [1]. The present data article adds significant value to the published research paper by providing a comprehensive and publicly available resource for training and testing the proposed modal-amodal segmentation algorithm. Researchers can utilize this dataset to replicate experiments, validate findings, and further advance the field of fruit detection and sizing in orchard management.

## 3 Data Description

**Fig. 1:**
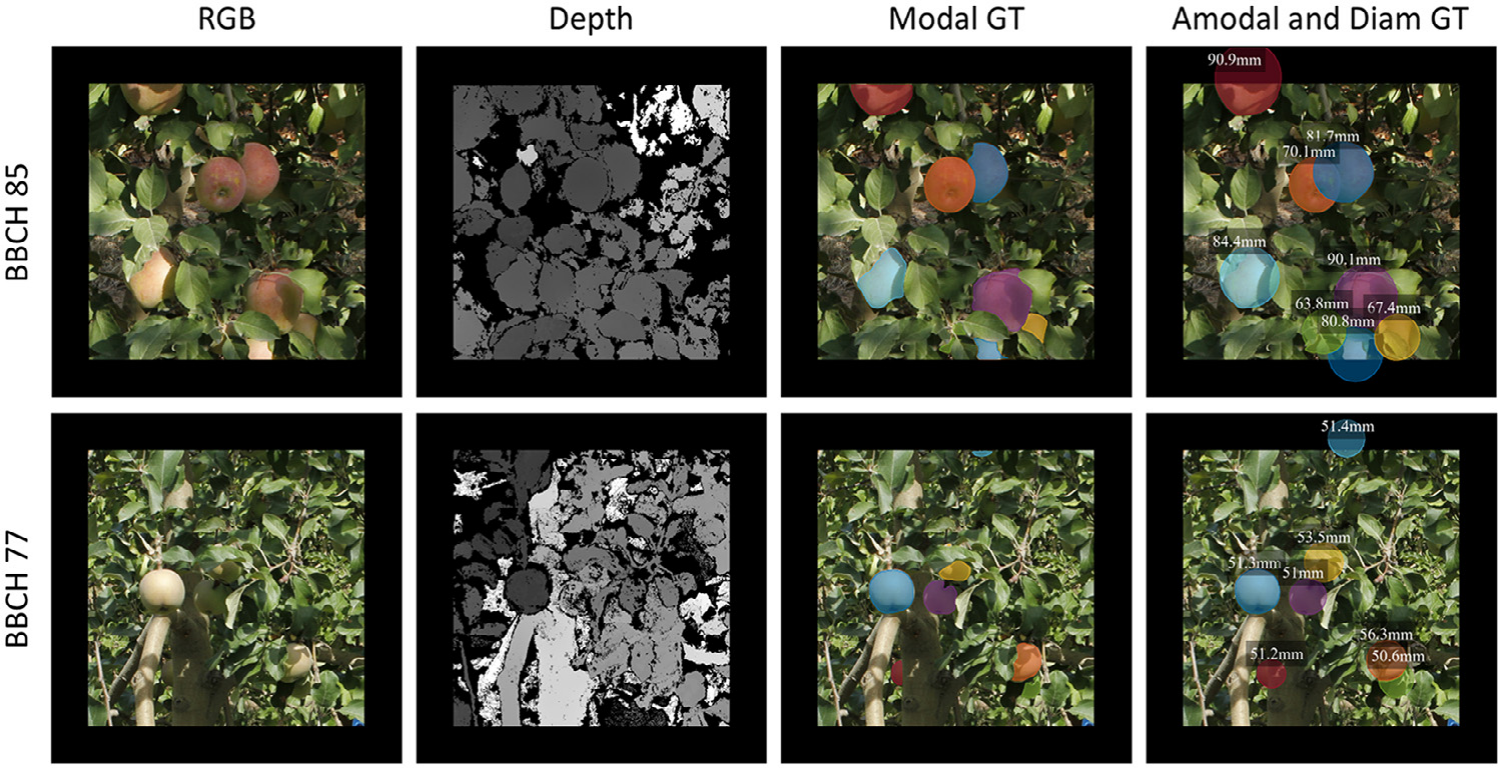
Fuji images and annotations. Each row corresponds to an image acquired at a different apple maturity stage: BBCH 85 and BBCH 77. The first column shows the RGB image, the second column displays the Depth image, the third column illustrates the modal segmentation masks ground truth, and the fourth column presents the amodal segmentation masks along with the apple diameters ground truth.

**Fig. 2:**
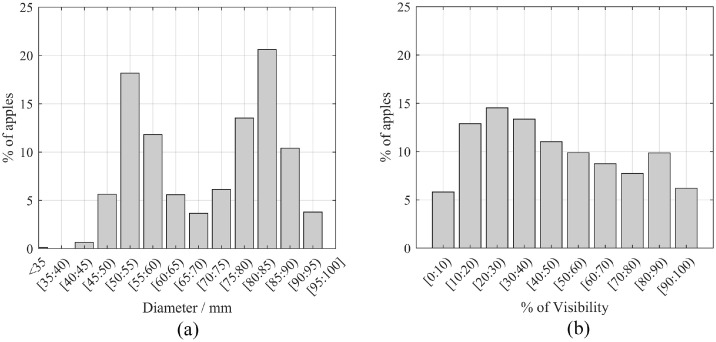
a) Bar plot showing the percentage of apples based on diameter. b) Bar plot showing the percentage of apples based on different levels of visibility.

This dataset comprises two distinct subsets of data. The first subset consists of 3925 RGB-D Fuji apple images, encompassing a total of 15,335 apple instances. Each apple instance is annotated with modal and amodal segmentation masks, as well as apple diameter ground truth. The modal mask represents the visible regions of apples, whereas the amodal mask includes both visible and occluded regions (Fig. 1). This subset is suitable for training, validating and testing fruit detection and sizing methods, as demonstrated in previous studies [1,4]. The availability of modal and amodal masks also enables the estimation of apples visibility, which is of interest in fruit sizing and harvesting robotics [7]. Fig. 2 shows the distribution of diameters and visibility of all annotated apples in this subset. The diameter distribution shows two peaks in the ranges of [50:55) mm and [80:85) mm, which correspond to the mean diameter of apples measured in the BBCH77 and BBCH85 growth stages [5] from which this data was acquired.

The second subset of data comprises 2731 RGB-D images captured in an Elstar apple orchard. These images were obtained from five apple trees on four different dates, corresponding to the apple growth stages of BBCH75, BBCH77, BBCH79 and BBCH85, in the BBCH growth scale [8]. This subset includes the mean diameter of apples on each tree at every growth stage (Table 1). It serves as a valuable resource for evaluation of fruit sizing methods trained with the aforementioned subset, under diverse data and environmental conditions different from those used during the training phase.

**Table 1:** Mean calliper of apples grown in the 5 imaged Elstar trees on the four different dates that data was collected. All diameter values are in millimetres.

Date	Tree 27	Tree 48	Tree 63	Tree 70	Tree78
21/06/19	36.05	44.39			
03/07/19	43.97	51.61	40.12	43.98	50.92
16/07/19	57.33	60.58	49.15	51.88	57.64
23/08/19	73.49	76.19	66.76	69.77	75.49

**Fig. 3:**
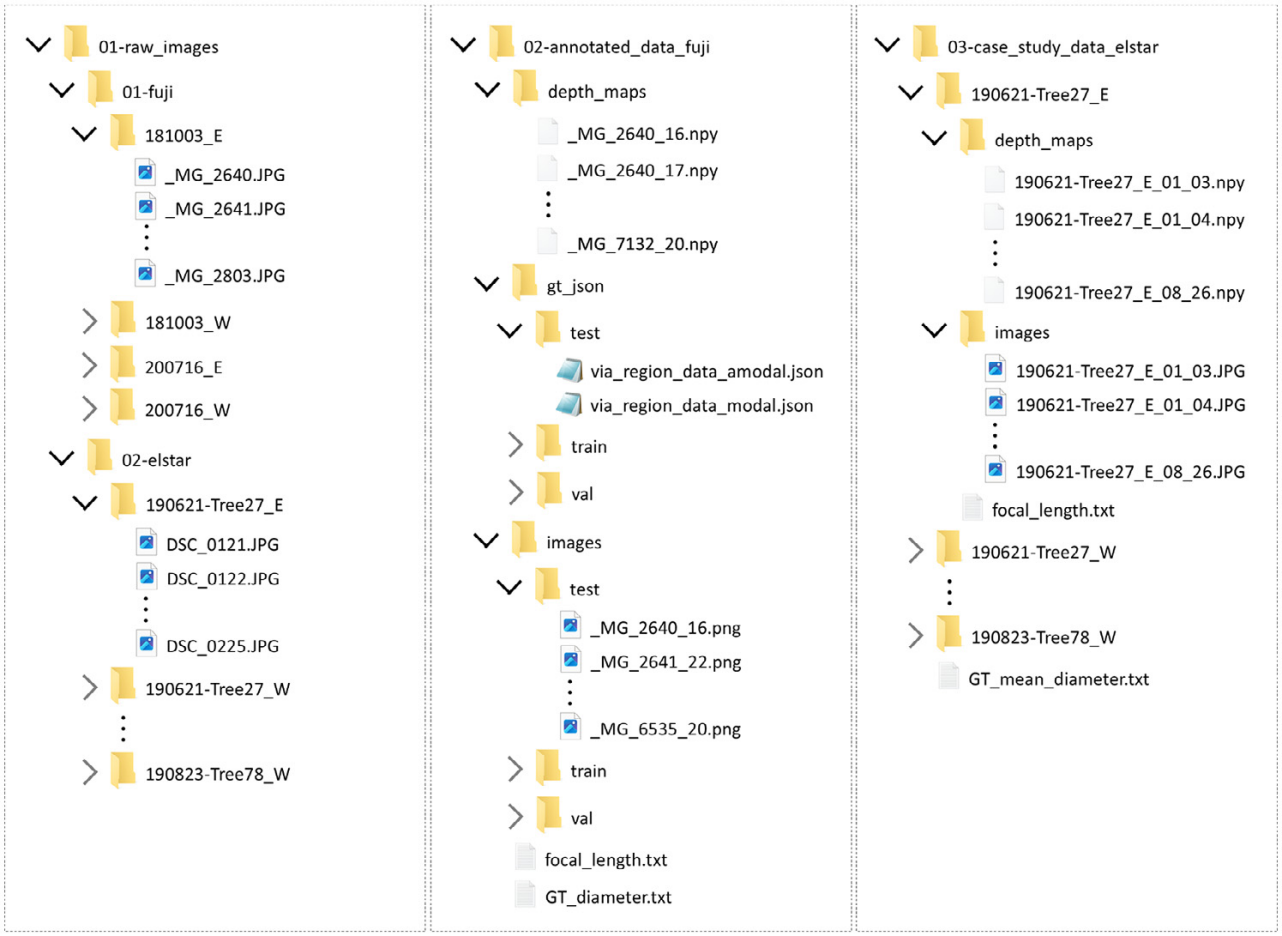
Dataset files and folder structure for each data subset: row data, annotated Fuji data and Elstar data.

The dataset can be downloaded at https://doi.org/10.34810/data916 [2]. Once it is unpacked, the dataset contains three main folders: ‘01-raw_images’; ‘02—annotated_data_fuji’; and ‘03-case_study_data_elstar’ (Fig. 3). The ‘01-raw_images’ directory contains all raw images used to build the dataset. The Fuji apple images are structured with subfolders depending on the date and side of the row of trees from which the images were captured. These subfolders are named with the following coding: YYMMDD_S, where YYMMDD refers to the date (year, month and day), and S refers to the side of the row of trees imaged (East and West). All raw Fuji images have a size of 5184 × 3456 pixels and were saved in JPG format. The raw Elstar apple images are structured with subfolders depending on the date, the tree number and the side of the row of trees that was imaged. These subfolders are named with the coding YYMMDD-TreeXX_S, where XX is the imaged tree number. All raw Elstar images have a size of 6000 × 4000 pixels and were saved in JPG format.

The ‘02-annotated_data_fuji’ directory contains all annotated Fuji RGB-D images. Images of this subset have a size of 1300 × 1300 pixels and were obtained by cropping raw image patches containing the Fuji apples that were manually measured in the field. This subset was split in training (2304 images), validation (814 images) and test (807 images) sets. Training images include images acquired from the west side, while validation and test images were acquired from the east side. This criterion was applied because raw consecutive images had a certain percentage of overlap. By using the images acquired from one side for training and from the other side for validation and testing, the authors aimed to ensure that there is no overlap between training and validation/test sets. The ‘depth_maps’ subfolder includes all image depth maps. These files have the same filename as the colour images but with NPY extension. These NPY files can be loaded in Python (recommended) or other programming languages (Matlab, C++, …) with the corresponding NumPy libraries or packages. Once loaded, they form a ndarray matrix of size 1300 × 1300 pixels (equal to colour image size) with float numbers corresponding to the depth values in meters. The modal and amodal segmentation masks are provided in the ‘gt_json’ subfolder with JSON extension files. These files can be loaded with Python (recommended) or other programming languages (matlab, C++, …) with the corresponding JSON libraries. Once the JSON file is loaded, it creates a dictionary of size equal to the number of images in the set. For each image, there is a subdictionary with ‘region’ attributes containing the segmentation mask annotations for each instance (apples) appearing in the image. The ‘shape_attributes’ includes the polygon shapes that encloses the masks, defined by its vertices points with x and y pixel coordinates, while the ‘apple_ID’ attribute specifies the identification number of each annotated instance. Finally, the focal length of all annotated images is provided in the ‘focal_length.txt’ file, while the diameter ground truth of all annotated apple_ID's is provided in millimeters in the ‘GT_diameter.txt’.

The ‘03-case_study_data_elstar’ directory contains the RGB-D Elstar images. Similar to the Fuji images, the images in this subset also have a size of 1300 × 1300 pixels and were obtained by cropping raw image patches. The depth maps are also saved in NPY extension and have the same size as the corresponding colour images. Besides the RGB-D images, this subset also provides information of the focal length of all images and the mean apples diameter measured on each tree at each measured date.

## 4 Experimental Design, Materials and Methods

**Fig. 4:**
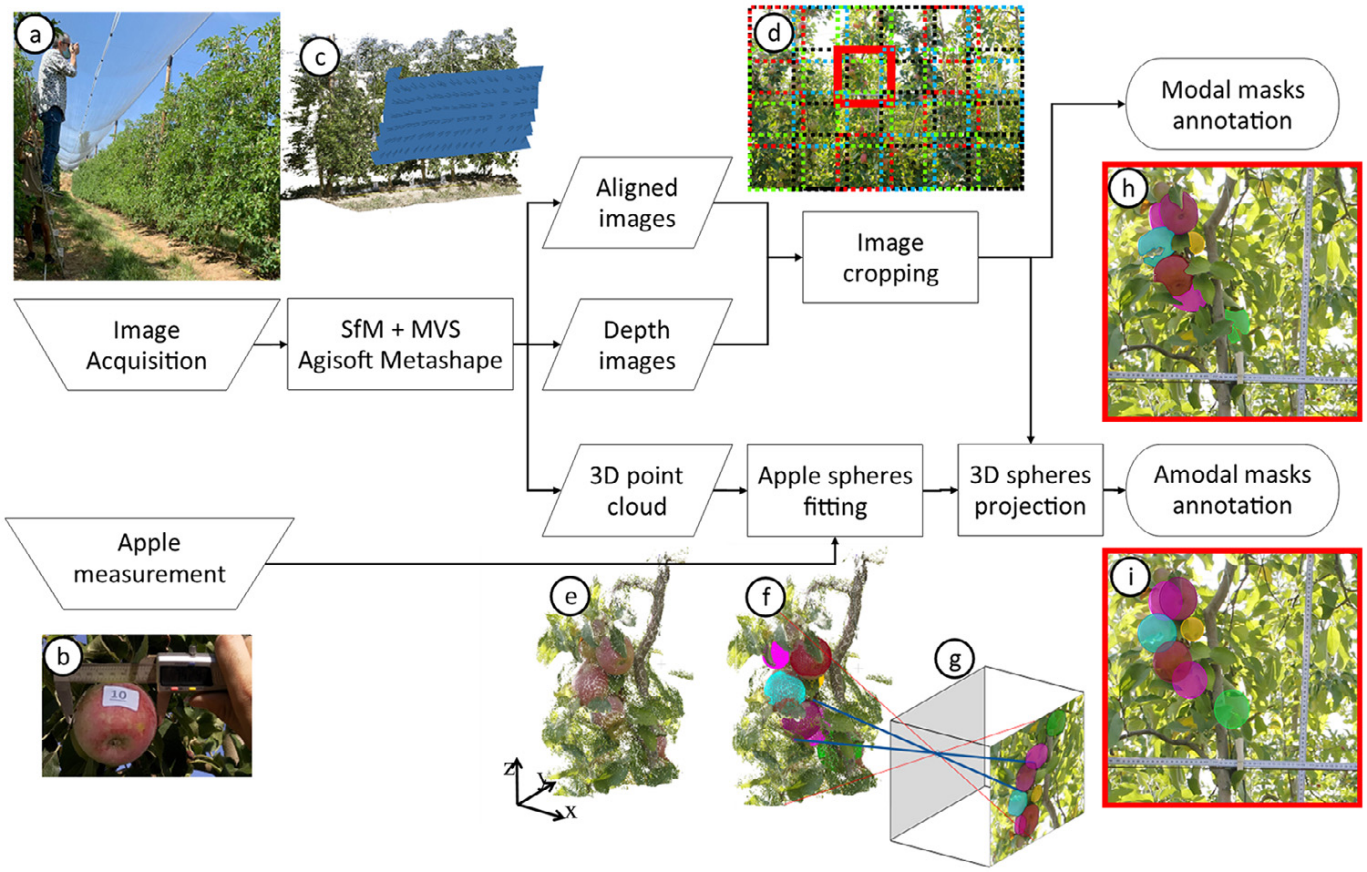
Data curation and annotation outline.

The methodology pipeline used to create and annotate the Fuji dataset is illustrated in Fig. 4. Raw images were captured in the field (Fig. 4a) following the procedure described in [9]. These images were acquired in a Fuji apple orchard situated in the province of Lleida, Catalonia, Spain. Fuji apple trees were photographed using a handheld EOS 60D DSLR camera (Canon Inc. Tokyo, Japan) on two different dates to ensure variability in apples diameters. The first data acquisition was carried out on October 3rd, 2018, when the apples were at the BBCH85 growth stage. The second data acquisition took place on July 16th, 2020, with the apples at the BBCH77 growth stage.

A total of 615 apples grown on the imaged trees were manually measured and annotated with an apple_ID. This measurement was carried out with a Vernier calliper by measuring the maximum horizontal diameter, as depicted in Fig. 4.b. The consecutive images had a substantial overlap (more than 75%) to facilitate the application of Structure-from-Motion (SfM) and Multi-View-Stero (MVS) techniques (Fig. 4.c). These photogrammetric techniques were applied using Agisoft Professional Metashape software (v1.6.4, Agisoft LLC, St. Petersburg, Russia). This software was instrumental in obtaining intrinsic and extrinsic camera parameters (aligned images), depth maps of the images, and a 3D point cloud of the imaged trees.

The images and the depth maps were cropped into 24 image patches of 1100 × 1100 pixels each (Fig. 4.d). This cropping was performed to enhance the ratio between apple size (in pixels) and image size, as the raw images contained numerous small apples in comparison to the overall image size. After cropping the raw images, the resulting image patches exhibited a more favourable apple/image size ratio for object detection neural networks, as recommended in [10].

The resulting image patches were annotated with modal masks (Fig. 4.h) using a semi-automatic annotation procedure. Initially, the Mask RCNN pretrained with the Fuji-SfM dataset [11] was used to generate initial modal segmentation masks. Subsequently, these annotations were manually revised and corrected using the VIA annotation software [12]. Additionally, all apple segmentation masks were associated with the corresponding apple_IDs to establish a link between the masks annotations and the respective ground truth apple diameters.

To derive the amodal segmentation masks, the 3D point clouds obtained from the SfM and MVS processes were used (Fig. 4.e). All measured apples were identified in the 3D point cloud, and spheres with diameters equal to the apple diameters were fitted to the 3D models of all apples (Fig. 4.f). This sphere fitting procedure was executed using CloudCompare software (Cloud Compare [GPL software] v2.9 Omnia). For each image patch, the 3D spheres of the apples within the image field of view were projected onto the image plane following the pinhole camera model (Fig. 4.g). As the spheres are considered complete representations of apples (including both visible and occluded parts), their projection onto the image plane resulted in the amodal segmentation masks (Fig. 4.i). To account for the possibility of amodal apple masks extending beyond image edges, the image patches were padded with 100 blank pixels around the images, as indicated by the black zone around images in Fig. 1.

Regarding the Elstar images, they were captured in an Elstar apple orchard located in Randwijk, the Netherlands. This set of images includes five different trees that were photographed at four dates (June 21st, July 3rd, July 16th, and August 23rd), corresponding to the growth stages of BBCH75, BBCH77, BBCH78 and BBCH85. The camera used was a Nikon Z6 camera. The diameter of apples grown in 10 marked fruit clusters per tree was measured with a Vernier calliper on all the dates the trees were imaged, providing the mean apple diameter per tree at each growth stage (refere to Table 1). The measurement dates corresponding to apple growth stages of 75 and 85 did not coincide with the date of the image data. Therefore, the mean diameter was obtained by interpolating the mean apples diameters between the two closest measurement dates: June 13th and 25th for the BBCH75, and August 16th and 27th for the BBCH85. Regarding to the image acquisition, the procedure to obtain the RGB-D images and the focal length of the Elstar apple set was identical to the procedure outlined for Fuji set, including steps **a** to **d** of the data curation methodology described in the preceding paragraphs and illustrated in Fig. 4.

## Limitations

None.

## Ethics Statement

Authors have read and follow the ethical requirements for publication in Data in Brief and confirm that the current work does not involve human subjects, animal experiments, or any data collected from social media platforms.

## Declaration of generative AI and AI-Assisted Technologies in the Writing Process

During the preparation of this work the author(s) used ChatGPT-3.5 to improve readability and language. After using this tool, the author(s) reviewed and edited the content as needed and take(s) full responsibility for the content of the publication.

## CRediT authorship contribution statement

**Jordi Gené-Mola:** Conceptualization, Methodology, Investigation, Data curation, Writing – original draft, Visualization, Supervision. **Mar Ferrer-Ferrer:** Methodology, Data curation, Writing – review & editing. **Jochen Hemming:** Supervision, Funding acquisition, Writing – review & editing. **Pieter van Dalfsen:** Methodology, Investigation, Data curation, Writing – review & editing. **Dirk de Hoog:** Methodology, Investigation, Data curation, Writing – review & editing. **Ricardo Sanz-Cortiella:** Investigation, Resources, Data curation, Writing – review & editing. **Joan R. Rosell-Polo:** Investigation, Supervision, Project administration, Funding acquisition, Writing – review & editing. **Josep-Ramon Morros:** Methodology, Supervision, Writing – review & editing. **Verónica Vilaplana:** Methodology, Supervision, Writing – review & editing. **Javier Ruiz-Hidalgo:** Methodology, Supervision, Writing – review & editing. **Eduard Gregorio:** Conceptualization, Methodology, Investigation, Supervision, Project administration, Funding acquisition, Writing – review & editing.

## Data Availability

AmodalAppleSize_RGB-D dataset (Original data) (Dataverse)
